# Diacylglycerol Kinases in T Cell Tolerance and Effector Function

**DOI:** 10.3389/fcell.2016.00130

**Published:** 2016-11-10

**Authors:** Shelley S. Chen, Zhiming Hu, Xiao-Ping Zhong

**Affiliations:** ^1^Division of Allergy and Immunology, Department of Pediatrics, Duke University Medical CenterDurham, NC, USA; ^2^Institute of Biotherapy, School of Biotechnology, Southern Medical UniversityGuangzhou, China; ^3^Department of Immunology, Duke University Medical CenterDurham, NC, USA; ^4^Hematologic Malignancies and Cellular Therapies Program, Duke Cancer Institute, Duke University Medical CenterDurham, NC, USA

**Keywords:** diacylglycerol kinase, regulatory T cells, invariant NKT cells

## Abstract

Diacylglycerol kinases (DGKs) are a family of enzymes that regulate the relative levels of diacylglycerol (DAG) and phosphatidic acid (PA) in cells by phosphorylating DAG to produce PA. Both DAG and PA are important second messengers cascading T cell receptor (TCR) signal by recruiting multiple effector molecules, such as RasGRP1, PKCθ, and mTOR. Studies have revealed important physiological functions of DGKs in the regulation of receptor signaling and the development and activation of immune cells. In this review, we will focus on recent progresses in our understanding of two DGK isoforms, α and ζ, in CD8 T effector and memory cell differentiation, regulatory T cell development and function, and invariant NKT cell development and effector lineage differentiation.

## Introduction

Diacylglycerol (DAG) and phosphatidic acid (PA) are two key lipid second messengers that facilitate efficient receptor-mediated signaling in immune cells along with many other cells. They regulate numerous intracellular signaling molecules to control cell differentiation, proliferation, survival, and function. Following T cell receptor (TCR) engagement, DAG is produced through the activation of Phospholipase Cγ1 (PLCγ1), which hydrolyzes membrane phosphatidylinositol bisphosphate (PIP_2_) to DAG and inositol trisphosphate (IP3). DAG, together with other signal events, recruits downstream effector molecules to the membrane through their C1 domains and allosterically activates these effectors, with protein kinase Cθ (PKCθ), Ras guanyl–releasing protein 1 (RasGRP1), protein kinase D (PKD), Munc13s, and chimaerins being important for T cell development and/or function (Krishna and Zhong, 2013a; Merida et al., 2015).

DAG plays an important role in recruiting PKCθ to the plasma membrane and immune synapse in T cells (Diaz-Flores et al., 2003; Carrasco and Merida, 2004). The activation of PKCθ leads to TCR-mediated NF-κB and mammalian/mechanistic target of rapamycin complex 1 (mTORC1) activation in T cells (Sun et al., 2000; Isakov and Altman, 2002; Hamilton et al., 2014), which affects key processes, including T cell activation and survival (Manicassamy et al., 2006; Hayashi and Altman, 2007), IL-2 production (Werlen et al., 1998), T_H_2 responses (Cannons et al., 2004; Marsland et al., 2004), T_H_17 responses (Kwon et al., 2012), invariant NKT (*i*NKT) cell development and activation (Schmidt-Supprian et al., 2004; Fang et al., 2012), and Treg development (Gupta et al., 2008; Barnes et al., 2009; Medoff et al., 2009).

Ras guanyl–releasing protein 1 (RasGRP1) is another downstream molecule that is recruited to the cytoplasm membrane by DAG (Jones et al., 2002; Carrasco and Merida, 2004). RasGRP1 promotes activation of Ras by exchanging GDP for GTP, leading to the activation of the RAF1-MEK1/2-ERK1/2 pathway (Ebinu et al., 1998; Dower et al., 2000; Roose et al., 2005). Additionally, RasGRP1-Ras-Erk1/2 pathway functions upstream for TCR-induced mTORC1, mTORC2, and PI3K activation in T cells (Gorentla et al., 2011). RasGRP1 plays an essential role in conventional αβ T cell development (Dower et al., 2000; Fuller et al., 2012), particularly for the selection of thymocytes that express weak TCR signals (Priatel et al., 2002) and for early iNKT cell development (Shen et al., 2011a). While RasGRP1 appears dispensable for overall γδT cell development, it ensures IL-17 expressing γδT17 lineage differentiation and TCR-induced γδT cell activation (Chen et al., 2012). More recently, it was also found that RasGRP1, together with RasGRP3, promotes early thymic precursor generation (Golec et al., 2016). Additionally, RasGRP1 may play a role in promoting antigen-induced CD8 cell expansion by lowering the threshold of T cell activation (Priatel et al., 2010).

PKDs are recruited by both DAG and DAG-activated PKCs. Upon stimulation, inactive PKDs translocate from the cytosol to the plasma membrane in response to membrane DAG production, where they are then activated by novel PKCs (Rozengurt et al., 2005; Spitaler et al., 2006). PKDs have been shown to exert different effects on VDJ recombination at the TCRβ locus and on CD4 and CD8 expression during T cell development based on their localization at the cytosol or plasma membrane (Marklund et al., 2003; Spitaler et al., 2006). Additionally, PKD2 acts as a sensitive digital amplifier of TCR engagement, enabling CD8 T cells to match the production of inflammatory cytokines to the quality and quantity of TCR ligands (Navarro et al., 2014).

Munc13 proteins are mammalian homologs of the *C. elegans* Unc13, which are important for neurotransmitter secretion (Brose and Rosenmund, 2002). Munc13-1, Munc13-2, and Munc13-3 isoforms bind to DAG with high affinity. The Munc13-4 isoform lacks a C1 domain (Koch et al., 2000; Shirakawa et al., 2004), but it is involved in granule maturation and exocytosis in NK cells and cytotoxic T lymphocytes (CTLs) (Feldmann et al., 2003; Menager et al., 2007), phagosomal maturation, and the killing of intracellular bacteria in neutrophils (Johnson et al., 2011; Monfregola et al., 2012). Deficiency of Munc13-4 causes primary immune deficiency in patients (Feldmann et al., 2003; Cichocki et al., 2014).

Chimaerins possess Rac-specific GTPase Activating Protein (GAP) activity (Caloca et al., 1999; Yang and Kazanietz, 2007). Chimaerin isoforms α2 and β2 are expressed at different levels in T cells and have been shown to translocate to the immune synapse and to both participate in TCR signaling and receive regulation from it (Caloca et al., 2008; Siliceo and Merida, 2009). Chimaerins have been found to inhibit TCR-mediated NFAT activation and DAG-dependent actin polymerization to regulate T cell adhesion and chemotaxis (Siliceo et al., 2006).

Phosphatidic acid (PA) is produced both by the activity of DAG kinases (DGKs) and by the phospholipase D (PLD) family of enzymes in T cells. DGKs phosphorylate DAG to convert it to PA, while PLDs mediate the hydrolysis of phosphatidylcholine (Jenkins and Frohman, 2005; Zhong et al., 2008). The removal of PA is mediated by lipins, which can turn off PA-mediated signaling through dephosphorylation, and they have been shown to regulate mast cell function in the immune system (Csaki and Reue, 2010; Shin et al., 2013b). Intracellular levels of PA change dynamically in response to environmental stimuli (Wang et al., 2006). The downstream effector molecules of PA include a multitude of kinases, such as mTOR (Chen and Fang, 2002), phosphatidylinositol-4-phosphate 5-kinase (PIP5K) (Galandrini et al., 2005; Jarquin-Pardo et al., 2007; Micucci et al., 2008; Cockcroft, 2009; Yoon et al., 2011), spingosine kinase (SPHK ½), RAF1 (Ghosh et al., 1996; Shome et al., 1997; Rizzo et al., 1999, 2000; Andresen et al., 2002), and other molecules, such as Src homology region 2 domain-containing phosphatase 1 (SHP1) (Frank et al., 1999), kinase suppressor of Ras 1 (KSR1, a scaffolding protein that interacts with several components of the Raf-MEK-ERK cascade) (Morrison, 2001; Kraft et al., 2008), and Sos, another guanine nucleotide exchange factor for Ras activation (Zhao et al., 2007). Both PLD and DGK-derived PA has been shown to directly activate mTOR in non-T cells (Chen and Fang, 2002; Avila-Flores et al., 2005). In these cells, PA can also activate mTOR indirectly via ERK (Winter et al., 2010), but such a mechanism has not been examined in T cells. In T cells, DGKα and ζ mainly inhibit TCR-induced mTOR signaling by negative control of DAG-mediated RasGRP1 and likely PKCθ activation (Gorentla et al., 2011; Hamilton et al., 2014). However, DGK-derived PA has been shown to promote T cell maturation in the thymus (Guo et al., 2008) and to regulate innate immune responses (Liu et al., 2007). Future studies should determine the direct downstream of the effector(s) of PA that mediate its functions in these immune cells.

The diverse and important functions of DAG—and PA-mediated signaling suggest their levels must be tightly controlled temporally and spatially. DGKs switch from DAG-mediated signals to PA-mediated signals to dynamically regulate downstream pathways in response to the engagement of the TCR and many other receptors (Merida et al., 2008; Cai et al., 2009; Zhong et al., 2011). In mammals, there are ten DGK isoforms encoded by different genes, some of which also contain splicing variants, adding complexity to this family of enzymes. All DGKs contain a kinase domain and at least two cysteine-rich C1 domains but differ in the homology of their other structural domains as well as their interaction with other biomolecules. Based on their structural distinction and homology, DGKs are classified into five types that may differ in subcellular localization, function, and regulation. The existence of multiple isoforms poses a significant challenge in studying the physiological roles of any specific isoforms in cellular development and functions due to functional redundancies, a fact demonstrated in conventional αβ T cell and iNKT cell development in mice deficient in both DGKα and DGKζ (Guo et al., 2008; Shen et al., 2011b). Of these ten isoforms, DGKα and DGKζ as well as DGKδ are the major isoforms expressed in T cells (Zhong et al., 2002; Olenchock et al., 2006a; Sakane et al., 2007). Both DGKα and ζ have been found to regulate multiple signaling pathways downstream from the TCR (Zhong et al., 2002, 2003; Sanjuan et al., 2003; Baldanzi et al., 2011; Gharbi et al., 2011; Gorentla et al., 2011), such as the RasGRP1-Ras-Erk1/2 pathway, the PKCθ-IKK-NFκB pathway, mTOR signaling (Gorentla et al., 2011), and MAP kinase-interacting serine/threonine kinase (Mnk) 1 and 2 signaling (Gorentla et al., 2013). They control T cell development (Outram et al., 2002; Guo et al., 2008; Almena et al., 2013), activation and anergy (Zhong et al., 2003; Olenchock et al., 2006a; Zha et al., 2006; Baldanzi et al., 2011), survival (Baldanzi et al., 2011; Ruffo et al., 2016), secretion (Alonso et al., 2007, 2011; Chauveau et al., 2014), and effector function (Shin et al., 2012; Yang et al., 2016b). Besides T cells, DGKζ also regulates the development, survival, and function of mast cells (Olenchock et al., 2006b), B cells (Wheeler et al., 2013), dendritic cells and macrophages (Liu et al., 2007), osteoclasts (Zamani et al., 2015), and NK cells (Yang et al., 2016a). Extensive reviews about DGKs in immune cells have been published recently (Merida et al., 2008, 2015; Zhong et al., 2008; Krishna and Zhong, 2013b). Here, we will focus on recent literature concerning DGKs in T cell tolerance, iNKT cell development and function, and CD8 T cell-mediated antimicrobial and antitumor immunity.

## DGKα and DGKζ in T cell tolerance

Clonal deletion of highly self-reactive T cells in the thymic medulla, generation of properly functioning regulatory T cells (Treg), and T cell anergy are among the most important mechanisms of T cell tolerance that prevent autoimmune diseases (Metzger and Anderson, 2011; Xing and Hogquist, 2012). Although DGKα and ζ synergistically promote T cell maturation from the CD4^+^CD8^+^ double positive (DP) to the CD4^+^CD8^−^ or CD4^−^CD8^+^ single positive (SP) stage, no direct evidence has implicated DGKα and ζ in interference with negative selection in establishing central tolerance (Guo et al., 2008).

Regulatory T cells generated in the thymus (tTregs) dominantly suppress T cells and other immune cells to prevent autoimmune diseases. However, they also negatively regulate antitumor and antipathogen immune responses. tTregs are derived from CD4 SP thymocytes in the thymic medulla after relatively strong but transient TCR-MHC/peptide engagement and signaling (Mahmud et al., 2014; Li and Rudensky, 2016). They express Foxp3, a key transcription factor that is critical for their development, maintenance, and function. TCR signaling is not only essential for tTreg generation but also required for tTreg homeostasis and function (Kim et al., 2009; Delpoux et al., 2014; Levine et al., 2014; Vahl et al., 2014). Multiple DAG-mediated signaling pathways are involved in tTreg development and function, indicated by the impaired tTreg development and function in mice deficient in either RasGRP1-Ras or PKCθ-IKK-NFκB signaling. Both NFκB and AP1 are involved in transcriptional activation of Foxp3 expression and possibly in regulating other tTreg properties (Schmidt-Supprian et al., 2004; Willoughby et al., 2007; Chen et al., 2008; Gupta et al., 2008; Barnes et al., 2009; Medoff et al., 2009). Both the percentage and number of tTregs in the CD4^+^ population are increased in DGKζ-deficient (but not DGKα-deficient) thymocytes and splenocytes, compared to wild-type (WT) controls (Table 1). Additionally, Foxp3^−^CD25^+^ cells within the CD4 SP thymocytes are increased in a DGKζ-deficient thymus, suggesting that DGKζ negatively controls early tTreg development. The inhibitory effect of DGKζ on tTreg development is found to be dependent on its negative control of the NFκB/c-Rel and RasGRP1-Ras-Erk pathways (Joshi et al., 2013; Schmidt et al., 2013). Of note are reports that DGKα and ζ manifest differential effects on TNFα-induced NFκB activation in tumor cells and fibroblasts, with DGKα positively regulating PKCζ-mediated p65/RelA at serine 311 residue (Yanagisawa et al., 2007; Kai et al., 2009), while DGKζ inhibits TNFα-induced NFκB activation via decreasing NFkB phosphorylation at Ser468/536, its nuclear localization, and its association with CBP (Tsuchiya et al., 2015). It would be interesting to investigate whether such mechanisms also operate in T cells or downstream of TCR to contribute to DGKα and ζ function in tTreg differentiation. It also remains unclear if DGKα and ζ act redundantly or synergistically to control Treg differentiation and function.

**Table 1:** **Comparison of DGKα^**−/−**^, DGKζ^**−/−**^, and DGKα^**−/−**^ζ^**−/−**^ mice**.

		**DGKζ^−/−^**	**DGKα^−/−^**	**DGKα^−/−^ζ^−/−^**	**References**
T cell development	Positive selection	Not affected	Not affected	Severe decreases of CD4 SP and CD8 SP thymocytes	Zhong et al., 2003; Olenchock et al., 2006a; Guo et al., 2008
	Negative selection	Not affected	Not affected	Not affected	Guo et al., 2008
Regulatory T cell	Foxp3^−^CD25^+^ CD4^+^SP thymocytes	Increased frequencies	Increased but less obvious than DGKζ^−/−^	Not reported	Joshi et al., 2013; Schmidt et al., 2013
	Foxp3^+^ Treg	Increased in thymus and spleen	Not increased	Not reported	Schmidt et al., 2013
	Suppressive function (*in vitro*)	Enhanced	Not obviously changed	Not reported	Schmidt et al., 2015
iNKT cells	iNKT cell numbers	Not affected	Not affected	Severely decreased	Shen et al., 2011b
	iNKT17 cell	Decreased in numbers due to extrinsic mechanisms	Not reported	Not reported	Wu et al., 2013
CD8 T cells	Primary responses to pathogens	Enhanced expansion and cytokine production in response to LCMV	Less obvious expansion than DGKζ^−/−^ but similar enhanced cytokine production in response to LCMV	Severely impaired in migration, expansion, and cytokine production in response to LM-Ova	Zhong et al., 2003; Shin et al., 2012; Yang et al., 2016b
	Memory responses	Decreased formation; impaired in expansion, enhanced IFNγ and TNFα production in recall responses to LCMV	Decreased formation; impaired in expansion (more severe than DGKζ^−/−^), enhanced IFNγ but not TNFα production in recall responses to LCMV	Impaired formation and maintenance; Decreased expansion but enhanced IFNγ and TNFα production in recall response to LM-Ova	Shin et al., 2012; Yang et al., 2016b
	Sensitivity to TGF-β	Decreased	Not reported	Not reported	Arumugam et al., 2015
	Anti-tumor immunity-OT1 T cells	Enhanced expansion and effector function; Enhanced tumor control	Not reported	Not reported	Riese et al., 2011, 2013
	Anti-tumor immunity-Meso-CAR T cells	Enhanced effector function	Enhanced effector function	Stronger effector function than DGKα or ζ single deficiency; Better tumor control	Riese et al., 2013

**Figure 1:**
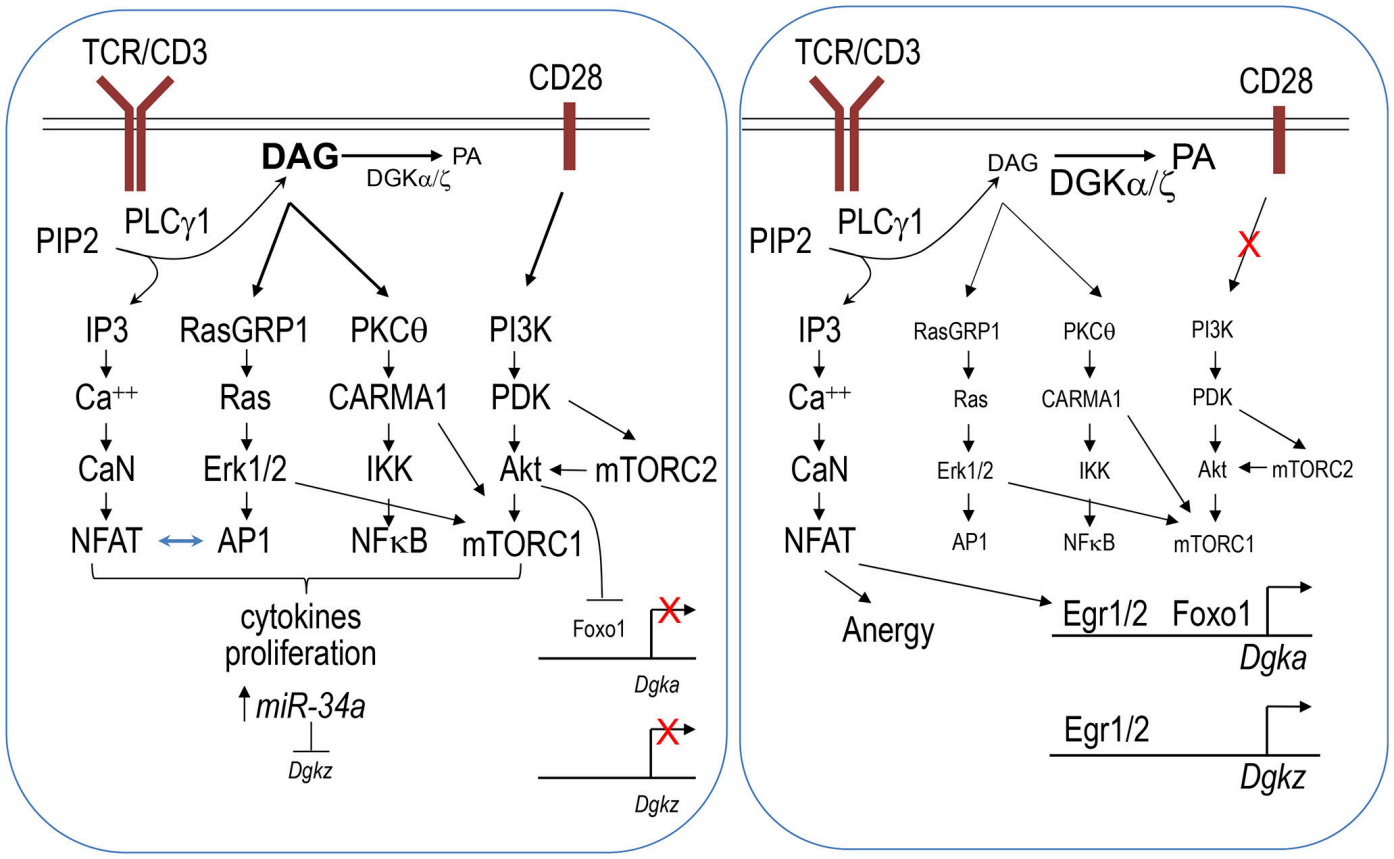
**DGKα and DGKζ in T cell activation and anergy**. Engagement of the TCR in the presence of co-stimulation results in strong activation of the PI3K-PDK1-Akt pathway (**left panel**). This pathway leads to mTORC2 signaling. Together with activation of the RasGRP1/Ras-Erk1/2 and PKCθ-CARMA1 pathways, they leads to mTORC1 activation. mTORC2 also promotes Akt activation via phosphorylation. Activated Akt phosphorylates Foxo1, leading to it sequestration in the cytosol and failure to activate DGKα transcription. In activated T cells, miR-34a is upregulated, which in turn downregulates DGKζ expression. Decreased DGKα and ζ expression leads to strong DAG-mediated signaling including increases of AP-1 and NFκB activity. AP-1 associates with NFAT to promote T cell activation. At the same time, AP-1 reduces monomeric NFAT to prevent it from inducing anergy promoting molecules. Strong DAG signaling together with IP3-CaN (calcineurin)-NFAT signaling allows full activation of T cells. In contrast, engagement of TCR in the absence of co-stimulation decreases PI3K-Akt-mTOR signaling, leading to increased nuclear Foxo1 and DGKα transcription (**right panel**). miR-34a mediated repression of DGKζ might also be lost under anergy inducing conditions. Increased DGKα and ζ expression may lead to a skewed balance between IP3 and DAG toward strong or selective Ca^++^-NFAT signaling and induction of Egr1/2, which further induce transcription of DGKα and DGKζ as well as other anergy promoting molecules. Selective IP3-Ca^++^-NFAT signaling in the presence of weak DAG-mediated signaling induces T cells to enter an anergic state.

T cell anergy is a form of peripheral tolerance whereby T cells that recognize self-antigens in the absence of co-stimulatory signals are rendered functionally inactive (Schwartz, 2003; Powell, 2006; Fathman and Lineberry, 2007; Chappert and Schwartz, 2010; Kalekar et al., 2016). In anergic T cells, DAG-mediated signaling, including Ras/Erk1/2, NFκB, and mTOR activation, is diminished, while Ca^++^-mediated signaling and NFAT are selectively elevated or unhindered (Powell, 2006; Chappert and Schwartz, 2010; Xie et al., 2012; Figure 1). Both DGKα and ζ are expressed at higher levels in anergic T cells than in activated T cells (Macian et al., 2002; Olenchock et al., 2006a; Zha et al., 2006). Deficiency of either DGKα or ζ or inhibition of DGK activity contributes T cell resistance to anergic induction (Olenchock et al., 2006a; Zha et al., 2006), while overexpression of DGKα promotes T cell anergy (Zha et al., 2006). Because DAG and IP3 are produced at an equimolar ratio by PLCγ1 from PIP2, the elevated DGKα and ζ expression in anergic T cells may shift the equilibrium of IP3 and DAG toward the predominance of IP3-Ca^++^-NFAT signaling over DAG signaling and subsequent AP1 induction. NFAT forms a NFAT/AP1 dimer to promote T cell activation, but it also functions as a monomer to induce transcription of anergy-promoting molecules, such as Cbl-b and TRAIL (Macian et al., 2002; Wu et al., 2006). It is postulated that elevated DGK activity may lead to NFAT monomer predominance over NFAT/AP1 dimer for anergy induction (Zhong et al., 2008; Krishna and Zhong, 2013a), although experimental evidence has not yet been presented.

An important issue is how DGKα and ζ expression is regulated. The transcription factor early growth response gene 2 (Egr2) is upregulated in anergic T cells and plays an important role in T cell anergy (Zheng et al., 2012). It binds directly to both *Dgka* and *Dgkz* promoters to increase the expression of these genes as well as several other anergy-promoting genes (Zheng et al., 2012, 2013). Another transcription factor, Foxo1, also directly promotes *Dgka* transcription (Martinez-Moreno et al., 2012). Foxo1 function, which is regulated by its subcellular localization between the cytosol and nuclei, is sequestered in the cytosolic compartment following Akt-mediated phosphorylation, which prevents it from association with target genes. In naïve or unstimulated T cells, nuclear Foxo1 activates *Dgka* expression. TCR engagement in the presence of CD28 costimulation induces strong PI3K/Akt activation, which may reduce nuclear Foxo1 and subsequent DGKα expression to ensure full T cell activation and avoidance of anergy (Martinez-Moreno et al., 2012). DGKζ expression has also been found to be regulated by microRNA. Two conserved sequences that match to the miR-34a seed sequence are located in the coding region and 3′ untranslated region (3′ UTR) of *Dgkz*. miR-34a expression is greatly upregulated in activated T cells. miR-34a directly represses DGKζ expression through targeting both *Dgkz* 3′ UTR and the coding region to promote T cell activation (Shin et al., 2013a).

## DGKs in iNKT cell development and function

Invariant NKT (iNKT) cells express the invariant Vα14Jα18 TCR, which recognizes lipid antigens presented by MHC class I-like CD1d molecules (Kawano et al., 1997; Mendiratta et al., 1997; Gapin et al., 2001). They are derived from a unique innate-like lymphoid cell lineage and can rapidly respond to agonist stimulation in both innate and adaptive immune responses via production of cytokines, such as IL-4, IL-17, IL-10, IL-13, IFNγ, and TNFα (Bendelac et al., 2007; Coquet et al., 2008; Godfrey et al., 2010; Milpied et al., 2011; Brennan et al., 2013; Salio et al., 2014). iNKT cells participate in host defense against microbial infection, antitumor immunity, and many diseases, such as allergies, asthma, graft-vs.-host disease, and obesity (Osman et al., 2000; Terashima et al., 2008; Van Kaer et al., 2013; Berzins and Ritchie, 2014).

**Figure 2:**
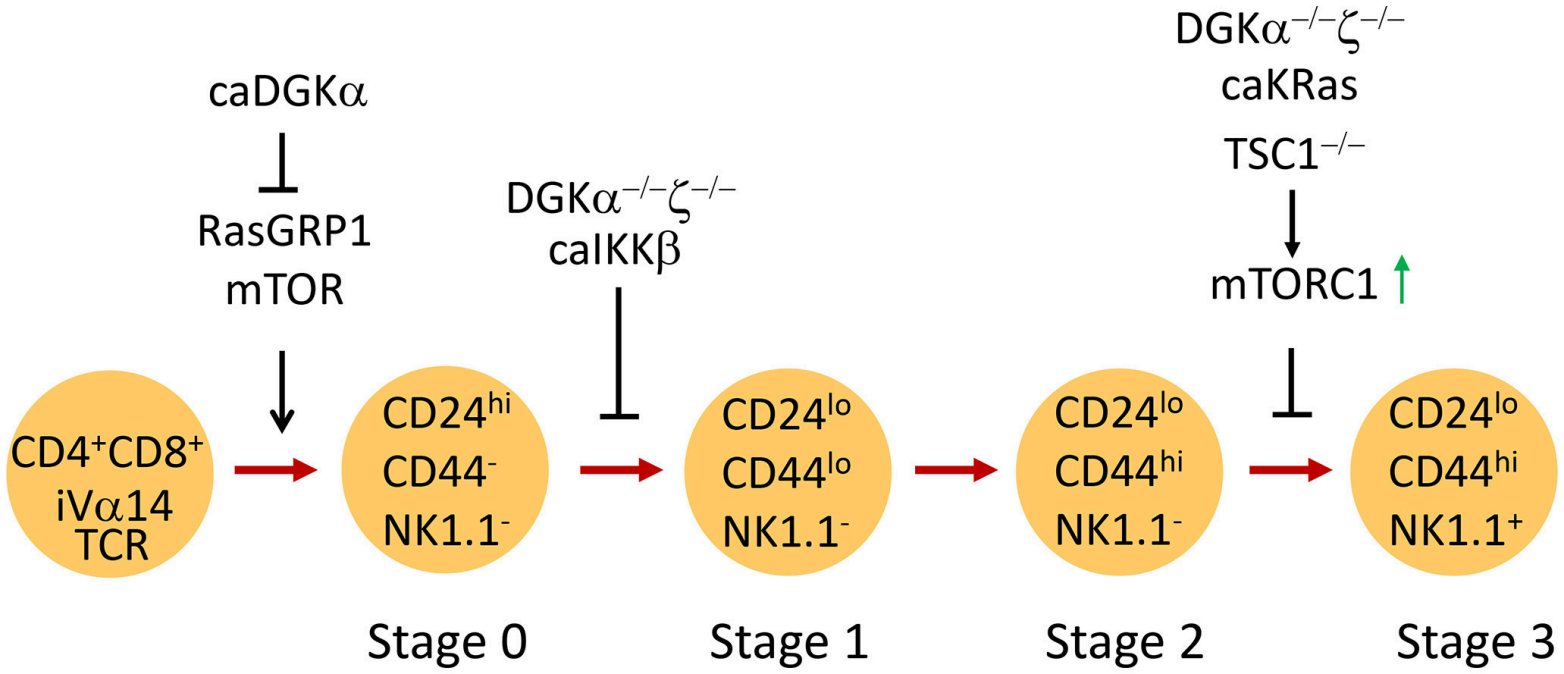
**Regulation of iNKT cell development by DGKα and DGKζ**. CD4^+^CD8^+^ DP thymocytes expressing the *i*Vα14TCR undergo positive selection to become iNKT cells. RasGRP1/mTOR signaling is critical for generation of stage 0 iNKT cells. Constitutive DGKα inhibits iNKT generation possibly by inhibiting RasGRP1/Erk1/2 activation. DGKα and ζ double deficiency or overactivation of IKKβ causes similar blockade of early iNKT cell development. Overactivation of mTORC1 due to TSC1 deficiency leads to blockade of iNKT terminal maturation. DGKα and ζ double deficiency or expression of a constitutively active KRas also results in impaired iNKT terminal maturation, correlated with elevated mTORC1 activation.

Based on surface CD24, CD44, and NK1.1 expression, iNKT cells are traditionally defined by four developmental stages in the thymus: stage 0 (CD24^+^CD44^−^NK1.1^−^), stage 1 (CD24^−^CD44^−^NK1.1^−^), stage 2 (CD24^−^CD44^+^NK1.1^−^), and stage 3 (CD24^−^CD44^+^NK1.1^+^) (Bendelac et al., 2007; Godfrey et al., 2010; Figure 2). Recently, iNKT cells have also been defined into multiple terminally differentiated effector lineages, such as IFN-γ-producing iNKT1, IL-4-producing iNKT2, and IL-17-producing iNKT17 lineage (Matsuda et al., 2006; Michel et al., 2007, 2008). In addition, IL-10-producing iNKT10, T follicular helper (Tfh)-like iNKT cells (iNKT_FH_), and regulatory T cell (Treg)-like iNKT cells have also recently been described (Chang et al., 2012; Tonti et al., 2012; Sag et al., 2014; Lynch et al., 2015; Rampuria and Lang, 2015). iNKT1 and iNKT17 cells mostly reside in the CD44^+^NK1.1^+^ and the CD44^+^NK1.1^−^ICOS^+^ populations, respectively (Watarai et al., 2012; Constantinides and Bendelac, 2013; Lee et al., 2013; Wu et al., 2014b).

Both the RasGRP1-Ras-Erk1/2 and PKCθ-IKK-NFκB pathways have been shown to play important roles in iNTK cell development (Yang et al., 2015). Although it was initially thought that Ras and Erk1/2 activation were dispensable for iNKT cell ontogeny, two recent studies have provided evidence that the RasGRP1-Ras-Mek1/2-Erk1/2 pathway is critical for early iNKT cell development (Hu et al., 2011; Shen et al., 2011a). In RasGRP1-deficient mice, stage 0 iNKT cells as well as total iNKT cell count are significantly decreased, suggesting defective positive selection (Shen et al., 2011a). In concordance with these observations, mice expressing dominant negative Ras in developing thymocytes demonstrated iNKT cell developmental defects (Hu et al., 2011). The RasGRP1-Ras-Erk1/2 pathway activates mTORC1 and mTORC2 signaling as well as Mnk1/2 in developing thymocytes (Gorentla et al., 2011, 2013). Both mTORC1 and mTORC2, but not Mnk1/2, are important for early iNKT cell development (Gorentla et al., 2013; Shin et al., 2014; Wei et al., 2014; Zhang et al., 2014; Prevot et al., 2015), revealing a RasGRP1-Ras-Erk1/2-mTOR signal cascade in iNKT cells for their development. mTORC1, but not mTORC2, promotes PLZF nuclear localization, which may ensure iNKT cell maturation in stage 1 and differentiation to cytokine-producing cells (Shin et al., 2014; Prevot et al., 2015). In iNKT cells, both the DAG and the SLAM (signaling lymphocytic-activation molecule)-SAP (SLAM adaptor protein)-FynT pathway are involved in PKCθ and subsequent NFκB activation. The PKCθ-IKK-NFκB pathway is essential in the ontogeny of iNKT cells, at least in part by increasing expression of antiapoptotic proteins, such as Bcl-xL (Elewaut et al., 2003; Sivakumar et al., 2003; Schmidt-Supprian et al., 2004; Stanic et al., 2004; Chung et al., 2005; Nichols et al., 2005; Pasquier et al., 2005; Griewank et al., 2007; Fang et al., 2012), but it is independent of CARMA1 and Malt1 (Mucosa-associated lymphoid tissue lymphoma translocation protein 1) (Medoff et al., 2009). CARMA1 contributes to TCR-induced mTORC1 activation in T cells (Hamilton et al., 2014). Given the minimal requirement of CARMA1 for iNKT cell development, it would be interesting to determine if TCR-induced mTORC1 activation in iNKT cells would be independent of CARMA1.

Emerging evidence demonstrates that tight regulation of DAG-mediated signaling by DGK activity is critical for the development of iNKT cells. Elevated DGKα activity brought about by expressing a membrane-targeted caDGKα in thymocytes under the control of the proximal Lck promoter caused reduced Erk1/2 activation in thymocytes and a 50% decrease of thymic iNKT cells (Almena et al., 2013). Germline deletion of either DGKα or ζ did not significantly alter iNKT cell numbers in mice. However, simultaneous ablation of both enzymes resulted in a drastic decrease in the number of iNKT cells in the thymus and in peripheral lymphoid organs (Shen et al., 2011b), correlated with prolonged DAG accumulation, elevated Ras-Erk1/2 and PKCθ-IKK signaling, and enhanced activation of both mTORC1 and mTORC2 activities in DP thymocytes (Guo et al., 2008; Gorentla et al., 2011). In DGKα and ζ double knockout mice, there was a decrease in the number of stage 1 to stage 3 iNKT cells. Stage 0 iNKT cells were not examined. The remaining iNKT cells in these mice were mostly CD44^+^NK1.1^−^ stage 2 cells, suggesting that DGKα and ζ promote both early and terminal iNKT cell maturation (Shen et al., 2011b). Interestingly, expression of constitutive active (CA) IKKβ in developing thymocytes caused a severe reduction in the number of stage 1–3 iNKT cells. Thus, DGKα and ζ double deficiency may cause dysregulation of the PKCθ-IKK-NFκB pathway, leading to early iNKT cell developmental blockage. Different from CA-IKKβ, expression of CA-KRas in thymocytes caused a selective blockage of the transition from stage 2 to 3 of iNKT cells and was associated with decreased T-bet expression (Shen et al., 2011b). Because CA-KRas and DGKα and ζ double deficiency caused elevated mTORC1 signaling (Gorentla et al., 2011) and overactivation of mTORC1 in the absence of TSC1 also resulted in a similar iNKT cell terminal maturation defect (Wu et al., 2014b), DGKα and ζ may synergistically promote iNKT cell terminal maturation at least in part by preventing overactivation of the RasGRP1-Ras-Erk1/2-mTORC1 signaling cascade.

The role of DGKs in iNKT effector functions, however, is less clear. DAG-mediated signaling pathways play important roles in T cell activation, effector lineage differentiation, and tolerance (Chen et al., 2012). They are thus expected to be important in iNKT activation and function. For example, PKCθ is essential for iNKT-mediated liver inflammation (Fang et al., 2012). In germline DGKζ-deficient mice, iNKT17, but not iNKT1 cell number, was selectively decreased. Interestingly, iNKT-17 defects caused by DGKζ deficiency can be corrected in chimeric mice reconstituted with mixed WT and DGKζ-deficient bone marrow cells, suggesting that DGKζ controls iNKT-17 differentiation via an extrinsic mechanism (Wu et al., 2013). Future investigation should define the type of cells that provide such a DGKζ-regulated extrinsic control of iNKT-17 development. Additionally, mTORC1 deficient iNKT cells are defective in activation and are not able to inflict liver damage (Shin et al., 2014). Overactivation of mTORC1 due to TSC1 deficiency shapes iNKT cell effector lineage fates and contributes to their resistance to anergy and enhanced antitumor immunity (Wu et al., 2014a,b). Given the ability of DGKs in regulating mTOR and PKCθ signaling, future studies should determine if DGKs intrinsically regulate iNKT cell functions and effector lineage differentiation under steady state and in various pathologic conditions.

## DGKα and ζ in CD8 T cell-mediated antipathogen immune responses

CD8 T cells play important roles in immune responses against pathogens, particularly intracellular pathogens. Upon microbial infection, naïve CD8 T cells are activated after engagement of their TCRs with pathogen-derived peptides presented by antigen-presenting cells. They massively expand and differentiate into cytotoxic T cells that are equipped to kill pathogen-infected target cells and secrete proinflammatory cytokines. A typical antigen-specific CD8 T cell-mediated response includes an expansion phase in which CD8 cells proliferate rapidly and differentiate into effector cells, a contraction phase in which 90–95% of effector CD8 cells die due to apoptosis, and a memory maintenance phase in which the remaining 5–10% of cells are retained as fast-responding memory cells (Williams et al., 2006; Harty and Badovinac, 2008; Zhang and Bevan, 2011). During the expansion phase, effector CD8 T cells differentiate into short-lived effector cells (SLECs, CD127^low^KLRG1^hi^) and memory precursor effector cells (MPECs, CD127^hi^KLRG1^low^) (Kaech et al., 2003; Sarkar et al., 2008). SLECs produce high levels of cytokines but are prone to death, while MPECs have high potential to differentiate to long-lived memory cells.

Engagement of the TCR on naïve CD8 T cells provides a critical signal that initiates their activation and expansion. TCR signal strength and quality regulate both the magnitude of expansion and the effector fates of CD8 T cells (Zehn et al., 2009; Iborra et al., 2013; Marchingo et al., 2014; Fulton et al., 2015) through the Ras-Erk1/2-AP1 and PKCθ-IKK-NFκB signaling pathways (Sun et al., 2000; Priatel et al., 2002; Zhong et al., 2008; Merida et al., 2015). An initial study found that DGKζ-deficient mice mounted an enhanced antiviral immune response following lymphocytic choriomeningitis virus (LCMV) infection. These mice showed enhanced expansion of viral-specific effector CD4 and CD8 T cells that contained higher percentages of IFNγ-producing cells 7 days after LCMV infection, which resulted in a quicker clearance of the virus than in WT mice (Zhong et al., 2003). A subsequent study further revealed that DGKα and ζ differentially regulate effector and memory CD8 T cell differentiation. While a deficiency of either DGKα or ζ resulted in enhanced effector CD8 T cell expansion, it slightly decreased memory CD8 T cell formation and response to LCMV infection, which correlated with elevated mTORC1 signaling in these cells (Shin et al., 2012).

Although deficiency of either DGKα or ζ enhances antiviral immune responses, DGKα and ζ double deficiency actually caused severe impairment of CD8 T cell-mediated responses to *Listeria monocytogenes* (LM) infection (Yang et al., 2016b). In an ovalbumin (OVA) specific OT1 TCR transgenic model and newly generated floxed DGKζ conditional-deficient mice where DGKα and ζ activity can be selectively deleted in naïve and memory CD8 T cells, it was found that ablation of both DGKα and ζ, but not of the individual DGKα or ζ isoform, impaired primary CD8 T cell responses (Table 1). At the earliest hours after LM-OVA infection, DGKα and ζ double deficient CD8 T cells expressed decreased levels of chemokine receptors CCR4, CCR5, and CXCR3 and showed impaired migration to the draining lymph nodes (dLNs). Cells that migrated to the dLNs were compromised in their proliferative ability due to not yet defined mechanism(s). In contrast to this *in vivo* setting, DGKα and ζ double deficient CD8 T cells proliferated more vigorously than WT controls *in vitro* following antigen stimulation, suggesting that the defect in proliferation was not due to intrinsic defects. It would be interesting to determine if DGKα and ζ are involved in regulating T cell/APC engagement for initiation of T cell activation. As a consequence of impaired expansion of DGKα and ζ double deficient CD8 T cells during primary immune responses, formation of memory cells was severely decreased as well. In addition, DGKα and ζ double deficiency compromised memory CD8 T cell function in homeostasis. Ablation of DGKα and ζ in preformed memory CD8 T cells accelerated the decline of these cells due to increased death and decreased homeostatic proliferative renewal (Yang et al., 2016b).

In DGKα and ζ double deficient CD8 T cells, TCR-induced NFκB nuclear localization was surprisingly diminished, although nuclear NFκB was elevated before stimulation (Yang et al., 2016b). A similar situation was also observed in T cells expressing a constitutive active IKKβ. CD8 T cells expressing a constitutive active IKKβ are defective in expansion *in vivo* following LM-OVA infection and are impaired in TCR-induced nuclear NFκB translocation (Krishna et al., 2012). It is likely, then, that elevated DAG levels may lead to an increase of basal activation of the PKCθ-IKK-NFκB pathway, which may trigger a negative feedback inhibition for TCR-induced activation of this pathway. Further studies should illustrate the exact negative feedback mechanism caused by DGKα and ζ double deficiency and by overactivation of IKKβ.

One consequence of decreased NFκB activation in DGKα and ζ double deficient CD8 T cells was decreased miR-155 expression and, subsequently, increased SOCS1 expression (Yang et al., 2016b). miR-155 promotes expansion of effector CD8 T cells and generation of memory CD8 T cells by targeting SOCS1 expression to ensure signaling from the common γ (γc) chain cytokine receptors (Dudda et al., 2013; Gracias et al., 2013). Common γ chain receptor signaling is known to be critical for CD8 effector and memory responses (Becker et al., 2002; Kieper et al., 2002; Carrio et al., 2004; Bachmann et al., 2007; Cui and Kaech, 2010; Sandau et al., 2010; Feau et al., 2011; Boyman and Sprent, 2012; Van Der Windt et al., 2012; Starbeck-Miller et al., 2014; Cui et al., 2015); SOCS1 negatively controls signaling from these γc-chain cytokine receptors (Cornish et al., 2003). Overexpression of miR-155 restored signaling from these receptors in DGKα and ζ double deficient CD8 T cells and partially corrected their defective responses. The data identified a DGK-NFκB-miR-155-SOCS1 axis that bridges TCR and γc-chain cytokine signaling for robust CD8 T-cell primary and memory responses to bacterial infection (Yang et al., 2016b).

## DGKα and ζ regulate CD8 T cell and CAR-T cell mediated antitumor immunity

A tumor microenvironment suppresses T cell mediated antitumor immunity, rendering tumor-infiltrating T cells hyporesponsive or anergic (Abe and Macian, 2013; Crespo et al., 2013). DGKζ-deficient CD8 T cells contain elevated antitumor immunity. DGKζ-deficient mice subcutaneously injected with the EL-4 thymoma had reduced tumor burdens and increased tumor-specific proliferative CD8 effector T cells compared to WT controls (Riese et al., 2011, 2013). Both increased Erk1/2 activation and decreased sensitivity to the suppressive cytokine TGF-β in DGKζ-deficient CD8 T cells may be responsible for stronger activation and antitumor immunity (Arumugam et al., 2015).

Recently, chimeric antigen receptor (CAR) T cells (CAR-T cells) have demonstrated superior activity in tumor control and, in some cases, tumor eradication (Fesnak et al., 2016). However, CAR-T cells have manifested limited efficacy for solid tumors in that they are subjected to suppression by the local tumor environment and may become hyporesponsive or anergic. Such hyporesponsive or anergic tumor-infiltrating T cells or CAR-T cells show decreased Ras/Erk activation but elevated DGKα and ζ levels (Moon et al., 2014). Both type 1 and type 2 DGK inhibitors are capable of reversing such hyporesponsiveness in tumor-infiltrating CAR-T cells *ex vivo*, leading to increased cytotoxicity (Moon et al., 2014). Consistent with this finding, genetic ablation of DGKα, ζ, or both DGKα and ζ enhanced CD8 T cells transduced with a mesoCAR, a CAR with high affinity to the human tumor antigen mesothelin. DGKα and ζ single or double deficient mesoCAR-T cells produced elevated IFNγ production and demonstrated stronger antitumor cytotoxicity than WT controls, which correlated with reduced sensitivity to TGFβ and increased expression of FasL and TRAIL, ligands for the death receptors FAS and TRAIL-RI/RII. Importantly, DGK-deficient mesoCAR-T cells controlled mesothelioma *in vivo* better than WT controls (Riese et al., 2013). The enhancement of CAR-T function by DGKα and ζ double deficiency sharply contrasts with the defective anti-LM responses of DGKα and ζ double deficient CD8 T cells, suggesting differential requirements of DAG-mediated signaling downstream of CARs and TCR and for CAR-T and conventional CD8 T cell activation.

## Summary

Over the past few years, our understanding of the DGK family of enzymes in immune cells has been significantly advanced. DGKα and ζ act individually to negatively control T cell activation, effector CD8 T cell differentiation and function during antimicrobial and antitumor immune responses, and tTreg generation. DGKα and ζ also manifest functional redundancy in promoting conventional αβ T cell and iNKT cell development and in enhancing CAR-T cell function. The unexpected severe impairment of CD8 T cell-mediated immune responses to microbial infection in the absence of both DGKα and ζ underscores the importance of fine-tuning DAG levels and also suggests potential negative feedback mechanisms triggered by deregulated DAG-mediated signaling. Defining such mechanisms should shed additional light on the regulation of DAG-mediated signaling pathways. Additional efforts are also needed to illustrate the underlying mechanisms of differential effects of DGKα and ζ double deficiency on CD8 T cells during antitumor and antipathogen immune responses. While DGKα and ζ perform similar or redundant functions, a more prominent role of DGKζ than DGKα in certain aspects of T cell biology, such as effector CD8 T cell differentiation and Treg, development has been noted (Table 1); however, determinants of such differences between DGKα and ζ remain unclear. The drastic differences observed between DGKα and ζ double and single deficient CD8 T cells during immune responses beg for development of DGK isoform-specific inhibitors. Such inhibitors used individually or in combination may provide great advantages over pan-DGK inhibitors in modulating immune responses for therapeutic purposes in different disease settings to minimize undesirable side effects. Key elements, such as transcription factors, microRNAs, and posttranslational modifications that control the dynamic individual and synergistic functions of DGK isoforms in T cells are beginning to be appreciated and require further exploration for better understanding of their physiological importance and the development of novel strategies enabling selective modulation of DGK α and ζ expression and activities for treating autoimmune diseases, viral infections, and cancer.

## Author contributions

SC, ZH, and X-PZ are involved in preparation of the manuscript.

### Conflict of interest statement

The authors declare that the research was conducted in the absence of any commercial or financial relationships that could be construed as a potential conflict of interest.
